# Effects of High Concentrate-Induced Subacute Ruminal Acidosis Severity on Claw Health in First-Lactation Holstein Cows

**DOI:** 10.3390/ani13081418

**Published:** 2023-04-20

**Authors:** Johann Kofler, Michael Hoefler, Thomas Hartinger, Ezequias Castillo-Lopez, Johann Huber, Alexander Tichy, Nicole Reisinger, Qendrim Zebeli

**Affiliations:** 1 Department of Farm Animals and Veterinary Public Health, University Clinic for Ruminants, University of Veterinary Medicine Vienna, 1210 Vienna, Austria; 2 Christian-Doppler-Laboratory for Innovative Gut Health Concepts in Livestock (CDL-LiveGUT), Department for Farm Animals and Veterinary Public Health, Institute of Animal Nutrition and Functional Plant Compounds, University of Veterinary Medicine Vienna, 1210 Vienna, Austria; 3 Teaching Farm, VetFarm Kremesberg, University of Veterinary Medicine Vienna, 2563 Pottenstein, Austria; 4 Department of Biomedical Sciences, University of Veterinary Medicine, Platform for Bioinformatics and Biostatistics, 1210 Vienna, Austria; 5 DSM, BIOMIN Research Center, 3430 Tulln, Austria

**Keywords:** subacute ruminal acidosis, SARA day, early lactation, lameness, claw horn disruption lesions, claw health score, dairy cattle

## Abstract

**Simple Summary:**

In this study, 24 first-lactation Holstein cows were evaluated to investigate how different levels of diet-induced subacute rumen acidosis (SARA) during early lactation (up to 70 days in milk; DIM) affect claw health. Claw health was monitored by recording claw lesions at three hoof trimming visits (before calving, at 70 DIM, and three months thereafter), and by assessing locomotion scores (LCS) 1 to 5 at two-week intervals. An intraruminal sensor monitored pH continuously, enabling the determination of the number of days the cows experienced SARA (pH below 5.8 for more than 330 min in 24-h), which allowed us to derive a severity index for SARA. In this manner the cows were assigned to three groups retrospectively: light, moderate, and severe SARA. A statistically significant increase in lameness incidence was observed when comparing light and severe SARA groups, but no effect was observed on LCS and claw lesion prevalence.

**Abstract:**

This study aimed to evaluate the effects of diet-induced subacute rumen acidosis (SARA) severity during transition and the early lactation period on claw health in 24 first-lactation Holstein heifers. All heifers were fed a 30% concentrate (in dry matter) close-up ration three weeks before calving, then switched to a high-concentrate ration (60% dry matter), which was fed until the 70th day in milk (DIM) to induce SARA. Thereafter, all cows were fed the same post-SARA ration with around 36% concentrate in dry matter. Hoof trimming was performed before calving (visit 1), at 70 (visit 2) and at 160 DIM (visit 3). All claw lesions were recorded, and a Cow Claw Score (CCS) was calculated for each cow. Locomotion scores (LCS 1–5) were assessed at two-week intervals. Intraruminal sensors for continuous pH measurements were used to determine SARA (pH below 5.8 for more than 330 min in 24 h). The cluster analysis grouped the cows retrospectively into light (≤11%; *n* = 9), moderate (>11–<30%; *n* = 7), and severe (>30%; *n* = 8) SARA groups, based on the percentage of days individual cows experienced SARA. Statistically significant differences were found between SARA groups light and severe in terms of lameness incidence (*p* = 0.023), but not for LCS and claw lesion prevalence. Further, the analysis of maximum likelihood estimates revealed that for each day experiencing SARA, the likelihood of becoming lame increased by 2.52% (*p* = 0.0257). A significant increase in white line lesion prevalence was observed between visits 2 and 3 in the severe SARA group. The mean CCS in severe SARA group cows were higher at each visit compared to cows in the other two groups, but without statistical significance. Overall, this is the first study indicating that first-lactation cows fed a similar high-concentrate diet but with a higher severity of SARA tended to have poorer claw health, albeit with only partial statistical evidence.

## 1. Introduction

Lameness in dairy cows is frequently associated with painful claw lesions, which significantly affect animal welfare and production performance [1,2,3]. Claw disorders are responsible for approximately 85% of lameness in dairy cows [4]. However, not all claw lesions are associated with a clinically detectable lameness [1,5,6]. Based on their etiology, a distinction is made between (1) claw lesions resulting from excessive load to the corium from inside as a result of hemorrhages and bruising of the corium, when the pedal bone descends due to laminitis, and from outside, (2) infectious claw diseases, and (3) claw lesions/claw deformations resulting from excessive sole horn abrasion or predominantly from a distinctive genetic background [1,7,8,9,10]. Claw horn disruption lesions (CHDLs) include sole hemorrhages, double soles, concave dorsal walls, sole ulcers, toe ulcers, white line lesions/abscesses, and horn fissures, which can be caused by laminitis (subclinical, subacute, acute), but also by excessive load from outside due to prolonged standing time, overstocking, too long hoof trimming intervals, hard flooring surfaces, etc. [1,7,8,9,10,11,12].

Breeding progress in dairy cows over recent decades has led to a large increase in milk yield [13], which has tremendously increased animal energy and nutrient requirements. This is especially true during early lactation, when cows are typically fed diets rich in concentrates to support performance while alleviating the energy deficit. Yet, the benefits of large amounts of concentrates in the diet of dairy cattle come at the expense of physically effective fiber, which is necessary to maintain rumen health [14,15,16]. Not surprisingly, this feeding practice has been associated with an increased incidences of subacute rumen acidosis (SARA), and the latter has often been suggested to increase the risk of lameness in cows [17,18,19,20,21,22].

In particular, first-lactation cows experience greater metabolic and nutritional stresses during early lactation. These cows are burdened with their first parturition and being milked for the first time, and thus must consume much more dry matter (DM) to meet their increased energy demands. They are also frequently rehoused in a new group with older cows [11,15,23,24]. In addition, first-lactation cows are often not accustomed to a ration that contains high levels of rapidly fermentable carbohydrates such as starches and sugars. In heifers in late gestation, the rumen papillae are comparably shorter and less numerous, and the microbiome has a different composition than in multiparous cows [15,25,26,27]. Studies have also shown that primiparous cows have a longer-lasting drop in rumen pH than multiparous cows, resulting in a higher risk for developing SARA [27,28].

Research over recent years has repeatedly shown that even with the same high-concentrate diet, ruminal pH drops, and the SARA responses of cows are highly variable; so, cows show different susceptibilities to SARA [27,29]. SARA has been defined as occurring when the pH is less than 5.8 for longer than 330 min/d [28], and the frequency of this pH drop indicates the severity of SARA [14]. The severity of SARA has been linked to an increased release of endotoxins in the rumen [14], which after being translocated into the systemic circulation may enter the small dermal blood vessels of the claws, and induce circulatory disturbances, inflammation, and ischemia [8,30,31]. This event weakens the corio-epidermal suspension of the pedal bone in the horn capsule. Thus, the large body weight of the cow causes sinking of the pedal bone with associated hemorrhages and circumscribed bruising of the corium. This particularly occurs underneath the flexor tubercle. In the medium term, these processes result in impaired horn production and the development of CHDL and a concave dorsal wall [8,12,30,31,32].

The objective of this study was to evaluate the effects of the different severities of SARA induced by high-concentrate feeding during early lactation on various parameters of claw health in first-lactation Holstein cows. SARA severity rating was based on the number of days individual cows exhibit SARA as measured by continuous monitoring of their intraruminal pH by a sensor. The underlying hypothesis was that the severity of a high-concentrate diet-induced SARA (i.e., the number of days that different cows experienced SARA) will modulate the development of laminitis and thus result in the deterioration of claw health in first-lactation Holstein cows.

## 2. Materials and Methods

This study was part of a large research project approved by the Austrian Federal Ministry of Education, Science and Research (GZ: 2021-0.009.975). The present study was discussed and approved by the institutional ethics and animal welfare committee of the University of Veterinary Medicine Vienna in accordance with GSP guidelines and national legislation (ETK-037/03/2021).

### 2.1. Animals and Housing

The study was conducted on 24 preselected Holstein heifers of the same genetic background (confirmed by genotyping), age, and body weight (699 ± 81.5 kg BW and 27.1 ± 2.39 months of age at the first visit) for a larger research project, which was designed as a feeding trial conducted from three weeks before calving until 70 days in milk (DIM). For this study, the heifers were monitored for claw health starting from eight weeks before calving until 70 DIM, when the cows had finished the main feeding trial. The heifers calved in groups between March and September 2021, and the calving process proceeded without complications for all animals.

During the entire study period, the 24 animals were kept in a separated area of the loose housing barn of the research dairy farm of the Vetmeduni VetFarm (Pottenstein, Austria). There were 15 cubicles (each 2.6 m × 1.25 m) available, which were designed as deep pens with straw and manure mattresses with fresh straw bedding twice per week. The cubicle partitions were flexible and the “neck tube” was designed as a chain, which offered a clearance of approximately 25 cm. In addition to the 15 cubicles, the animals had free access to a large deep bedding area (15.7 m × 8.1 m) with a thick layer of long straw. The walkways were rubber-matted and cleaned of manure ten times per day using scrapers. The deep straw area was replenished twice a week with fresh straw, and it was also renewed every three weeks. For milking, the cows were driven about 50 m to the milking parlor twice per day via a concrete run. Foot baths were not used in this herd.

The feeding alley was equipped with feed troughs (RIC HOKO-Farm; Emmeloord, NL). The heifers calved in the deep straw area of the barn compartment. Prior to the start of the feeding study, all 24 pregnant heifers had been kept together for a period of five months in the separate dry cow barn of the VetFarm, which was also equipped with a large deep litter area and rubber mats at the feeding alley.

After the end of the feeding study at 70 DIM, the cows were integrated into the remainder of the herd at the farm where the walkways were equipped with rubber mats, and where the cows also had deep pens with straw and manure mattresses with fresh straw bedding twice per week. Further, the cows had access to a concrete run and to concrete floors in the milking parlor waiting area.

The average milk yield of the 24 Holstein cows during the first lactation was 10,500 kg, with 3.78% fat and 3.24% protein.

### 2.2. Feeding Regime

The research project’s feeding protocol was designed to feed heifers an all-forage diet during pregnancy and switch them to a close-up ration with a concentrate level of 30% in the DM as total mixed ration (TMR) three weeks before expected calving to provide enough energy and nutrients for the pregnant heifers [33].

After calving, all cows were fed an early lactation TMR, rich in concentrates (60% DM), to induce a SARA challenge. To avoid potential cases of acute acidosis, the proportion of concentrate was gradually increased from 30 to 60% within the first week postpartum. This high-concentrate diet was fed to all cows until 70 DIM and was termed the “SARA ration”. Thereafter, the SARA ration diet was discontinued, and cows were fed a TMR with 36% concentrate in the DM (termed post-SARA ration), as per normal feeding management regulations of the farm, to provide sufficient energy, physically effective fiber, and other nutrients. The ingredients and compositions of all diets are shown in Table 1.

The TMRs were prepared daily with a Trioliet feeding robot (Trioliet Feeding Technology, Oldenzaal, NL) and the ration was offered fresh to the cows twice daily at approximately 10:30 am and 4:00 pm to assure ad libitum intake, aiming at a target of 5–10% feed residuals. Feed refusals were collected in the morning before offering the new fresh feed and discarded.

### 2.3. Measurement of Intraruminal pH and Classification of Cows Based on SARA Severity

As part of the protocol of the original research project, the cows were administered a wireless rumen bolus (pH Plus Bolus SX-1042A, smaXtec^®^ animal care GmbH, Graz, Austria) approximately four weeks before their expected day of calving. This device continuously measured the rumen pH. Measurement data, which were transmitted continuously at 10-min intervals, enabled determination of the period during which the pH was below a threshold value of 5.8. If the pH was below 5.8 for more than 330 min within 24 h, that day was considered a SARA day [28]. The number of SARA days was related to the total duration of the feeding trial of 91 days (close-up plus SARA diet feeding).

Classification of SARA severity was performed retrospectively based on the percentage of the days the cows experienced SARA, as the duration of SARA has been related to its severity [14,28]. For the classification, the data of the percentage of the days with SARA underwent a cluster analysis using the procedures of distance (PROC DISTANCE) and cluster (PROC CLUSTER) in SAS (version 9.4, SAS Institute Inc., Cary, NC, USA). First, the procedure distance computed the distance matrix of the percentage days of SARA among cows, called the Euclidian distances. The proximity measure was computed using the respective standard deviation. Thereafter, the cluster procedure performed a Ward’s minimum-variance cluster analysis based on the distance matrix created before by the proc distance. The heights of the dendrograms were specified using the R^2^ method (0 to 1 with 1).

### 2.4. Locomotion Scoring

Starting two weeks before the expected date of parturition, all animals were subjected to a gait assessment at two-week intervals according to the locomotion scoring system of Sprecher et al. [34]. This was performed by one and the same trained evaluator. Relief posture, relief movement and the back line were assessed in each animal while standing and walking and scored to give a locomotion score (LCS) 1–5. LCS 1 corresponds to a bovine free of lameness and LCS 5 corresponds to a bovine that is not weightbearing a limb to any extent [34]. Locomotion scoring in cows was performed either while walking to the milking parlor or while walking in the separated barn area. The evaluator was blinded to ruminal pH and SARA data throughout the study.

### 2.5. Hoof Trimming Time Points and Documentation of Claw Lesions

All 24 heifers were subjected to a first functional hoof trimming episode eight weeks before the expected calving date. The second and third functional hoof trimmings were performed on all cows immediately after the end of feeding the SARA ration (at 70 DIM) and three months later (at 160 DIM). Cows exhibiting lameness (LCS ≥ 2) during the observation period were immediately examined and adequately treated by three trained veterinarians, who were blinded to the ruminal pH or the severity of SARA data. Evaluation of claw health at visit 3 (three months after the end of SARA ration) was performed to evaluate any potentially delayed effects of SARA or high-concentrate diet on claw health. During the post-SARA diet feeding, no further risk of SARA was expected [16].

All claw lesions observed during the three hoof trimming visits were electronically documented using the program “*Klauenmanager*” (SEG Informationstechnik GmbH, Bad Ischl, Austria). The program offers the capacity to calculate a claw health score (called “Cow Claw Score”, CCS) for each cow, which is determined by the integrated software from all documented claw lesions and their severity scores (score 1, 2 and 3) from all eight claws [35,36]. Definitions and descriptions of non-infectious claw lesions and infectious claw diseases and their three severity scores are integrated in the “*Klauenmanager*” program for all users, and they have also been published [35]. Table 2 displays the three severity scores of each claw lesion, except for certain single lesions, along with their assigned geometric severity scores. The severity scores were determined using internationally harmonized terminology based on the ICAR Claw Health Atlas and its appendices [37,38,39], as well as methods described in the previous literature [35,36,40,41], and recently extended by researchers from Berne, Switzerland (A. Steiner, C. Syring; personal communication).

### 2.6. Statistical Analyses

Most of the statistical analyses were performed with the program IBM SPSS^®^ Statistics for Windows, version 28.0 (IBM Corp., Armonk, NY, USA). For all documented claw lesions in these animals, the respective prevalence in percent at claw level was calculated as the proportion of affected claws to the sum of all claws from all cows within a SARA severity group. Mean, standard deviation, minimum and maximum values of LCS for each cow over the observation period were computed.

Lameness incidence was computed for each SARA group using the following formula: new cases of lameness at every gait assessment (*n* = 7) divided by the total number of eight cows per SARA group. Each lame cow at these time points in the two-week intervals was classified as a “new” case. The odds of various factors causing lameness based on the measured variables of SARA were tested with the logistic procedure (PROC LOGISTIC) of SAS (version 9.4, SAS Institute Inc., Cary, NC, USA). A model with a forward selection method was used to evaluate all potential influencing factors (i.e., dry matter intake of concentrate or overall dry matter intake), by selecting only those showing a significant effect (*p* < 0.05). Odds ratio (OR) estimates and the respective profile likelihood confidence intervals (CI) were evaluated. The UNITS statement was used to specify units of change for the continuous explanatory variable, enabling customized OR estimations. The analysis of maximum likelihood estimates was performed in Proc logistic to evaluate the effects of continuous explanatory variables (i.e., Wald Chi-Square test), and to compute the Wald CI and the predicted probability of lameness occurrence (i.e., using the option PLOTS = EFFECTS). The receiver operating characteristic (ROC) curve with the area under the curve (AUC) were also computed.

The comparison of the three SARA groups within the three hoof trimming visits in terms of the number of claws with claw lesions and means of LCS and lameness incidence was executed using Kruskal–Wallis H-tests and multiple comparisons with Bonferroni’s alpha error correction. The CSS were analyzed using a linear mixed effects model, with SARA group and hoof trimming visits as fixed effects and the animal as a random effect in the model. The evaluation of the CCS values in the SARA groups and regarding the three hoof trimming visits was carried out on the one hand in relation to each hoof lesion with the associated CCS, and on the other hand with the sum of all CCS values for each SARA group and each time point.

All multiple comparisons were performed using Sidak’s alpha correction procedure. The requirement of a normal distribution of data was proved with the Kolmogorov–Smirnov test. The proportions of observed lameness incidence within the three SARA groups were compared by applying the two-samples proportion test, performed using the program R (The R Foundation for Statistical Computing, Vienna, Austria). A *p*-value < 0.05 was considered significant.

## 3. Results

### 3.1. Number and Percentage of SARA Days, and SARA Severity Groups

**Figure 1:**
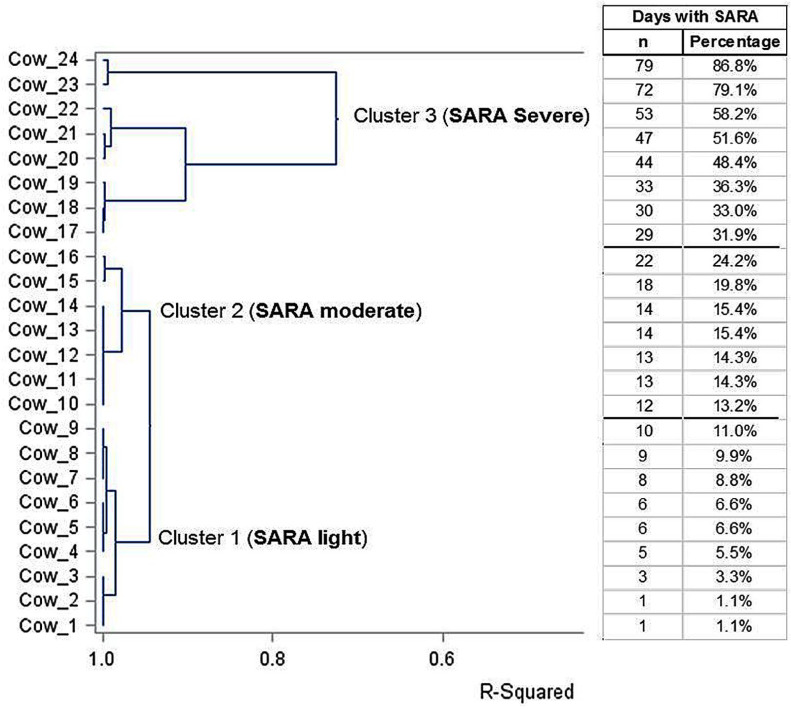
Results of the cluster analysis with the dendrogram and R^2^ of 24 Holstein cows clustered into three groups of SARA severity (light, moderate and severe) and the data of single cows regarding days experiencing SARA (as numbers or percentage of the days with SARA).

As anticipated, the number of days SARA experienced was very different among the first-lactation Holstein cows despite receiving the same high-concentrate ration. The cluster analysis performed retrospectively allocated the 24 cows into three SARA severity groups, namely, the SARA groups light, moderate and severe, with 9, 7 and 8 cows, respectively. The results of the cluster analysis with the three groups and their relative numbers of SARA days as assessed by the ruminal pH sensors are listed in Figure 1. The cluster analysis clearly clustered nine cows into the light SARA group with ≤11% of the days with SARA separately (R^2^ = 0.98). The moderate SARA group was clustered separately (R^2^ = 0.95) including seven cows with >11–<30% of the days with SARA, though this cluster was closer to the light SARA group than to the severe SARA group (Figure 1). Eight cows of the severe SARA group with >30% of the days with SARA built their own cluster, though with higher variation (R^2^ = 0.75) than the other two clusters. Furthermore, in the severe SARA group, two cows (i.e., 23 and 24) experienced SARA during the entire feeding time with 60% concentrate and two or nine days of the close-up period (79.1% and 86.8% of the experimental time under SARA; Figure 1).

### 3.2. Lameness Incidence and Locomotion Scores (LCS)

During the study period, a total of seven gait assessments starting from two weeks ante partum were performed in the 24 animals, resulting in a total of 168 gait assessments. Only LCS 1–3 were determined, and LCS 3 was detected only once (0.6%). LCS 2 was counted a total of twelve times, twice each in the light and moderate SARA groups and eight times in the severe SARA group. Lameness incidence in the 24 first-lactation cows during the entire observation period was 7.7% (±9.2). The minimum and maximum lameness prevalence values per gait assessment per SARA group were 0.0% and 25.0%, respectively. In total, cows in the light SARA group showed a lameness incidence of 3.2%, cows in the moderate SARA group had an incidence of 6.1%, and the highest lameness incidence of 14.3% was observed in cows of the severe SARA group.

Using lameness incidences as the basis for calculation, a statistically significant difference was found comparing the light SARA group with the severe SARA group cows (*p* = 0.023); the differences between the other groups were not statistically significant (light SARA group vs. moderate: *p* = 0.324; moderate SARA group vs. severe: *p* = 0.056). The mean LCS of cows in the individual SARA groups were low, ranging from 1.03 to 1.14 (Table 3). Using the LCS data, no statistically significant differences were detected between the three SARA groups at any hoof trimming visit (*p* = 0.229), but the highest difference was between the light SARA group and the severe SARA group (*p* = 0.095).

The logistic regression analysis with the forward selection method revealed that the factor days with SARA was the most significant factor associated with lameness in the cows of the present study, and not concentrate intake or dry matter intake. The analysis of maximum likelihood estimates and the Wald Chi-Square test predicted an increased (*p* = 0.0257) likelihood of cows suffering from lameness the longer the SARA was experienced, predicting that for each day under SARA, the likelihood of becoming lame increased by 2.52% (Wald 95% CI: 0.31–4.74%). Using 20, 40, and 60 as units of change for SARA days, the odd ratio analysis predicted an OR of 1.65 (95% Cl: 1.05–2.58) for every 20 days with SARA, and the OR increased to 2.74 (1.09–6.66) after 40 days, and further to 4.54 (1.52–17.23) after 60 days with SARA, all compared with 0 days with SARA. 

**Figure 2:**
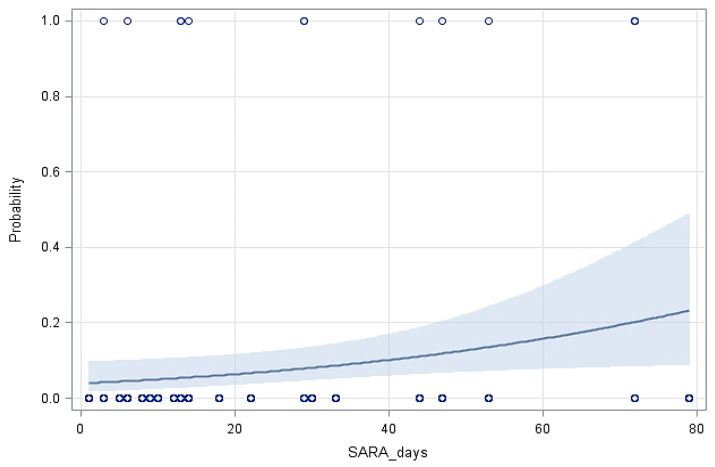
The predicted probability of lameness in cows with the 95% confidence limits (shaded area) based on the duration of SARA (Y = −3.1866 (±0.4834) + 0.0252 (±0.0113) X, *p* = 0.0257).

**Figure 3:**
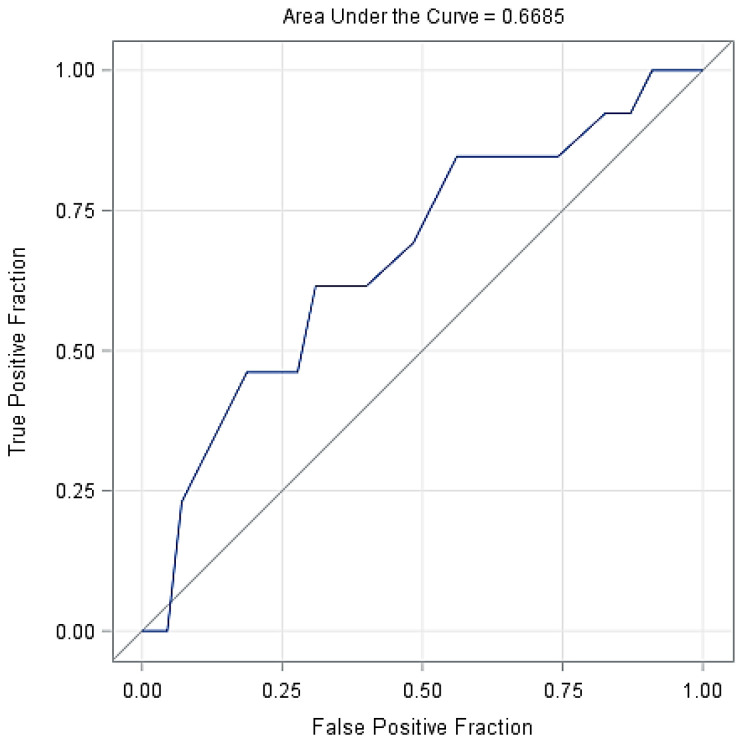
The receiver operating characteristic curve (ROC) of the model at various decision thresholds with the AUC of the model.

Figure 2 shows the probability of lameness in the cows based on the days with SARA, indicating that longer duration of SARA increased considerably the probability of cows becoming lame. Figure 3 shows the ROC curve at various decision thresholds and the AUC of the model. With around 66.9% AUC, the model has a satisfactory overall prediction power.

### 3.3. Type and Prevalence of Claw Lesions

The types and prevalences (at claw level) of claw lesions documented at the three hoof trimming visits in the three SARA groups are listed in Table 4. The causes of lameness episodes were double soles, white line lesions, acute digital dermatitis (DD-M2), and one single sole ulcer. This sole ulcer was diagnosed at hoof trimming before calving in a heifer in the severe SARA group. 

Concave dorsal walls of the claws of all cows in the three SARA groups were only documented at hoof trimming visit 2 (i.e., at the end of the high-concentrate feeding period at 70 DIM) with a mean prevalence of 3.1%, and at visit 3 with a mean prevalence ranging from 23.4% to 28.1% (Table 4). However, statistically significant differences regarding prevalences of concave dorsal walls across SARA groups were not detected, but statistically significant differences were observed between hoof trimming visits 2 and 3 in the light (*p* = 0.022), moderate (*p* = 0.032), and severe SARA groups (*p* = 0.010), and between visits 1 and 3 in all three SARA groups (*p* = 0.040 to 0.004).

The prevalence of sole hemorrhages increased among cows in SARA groups moderate and severe over the three hoof trimming visits, but without statistically significant differences. Double soles were diagnosed most frequently in cows in the light SARA group, and their prevalence increased (*p* = 0.042) from 0% before the start of the study to 12.5% three months after the end of high concentrate feeding (Table 4). Thus, there was a statistically significant difference in the prevalence of double soles between hoof trimming visits in the light SARA group (*p* = 0.042). However, no statistically significant difference in the prevalence of double soles was detected among the three SARA groups.

The mean prevalence of white line lesions (WLL) was 8.9% in the light SARA group, 14.0% in the moderate SARA group, and 12.5% in the severe SARA group before calving. At hoof trimming visit 3, the WLL prevalence was 12.5% in cows in the light SARA group and 18.7% in both other SARA groups (Table 4). The difference in the prevalence of WLL was statistically significant within the severe SARA group between hoof trimming visits 2 and 3 (*p* = 0.017), whereas there were no statistically significant differences between the three SARA groups at any time point.

The number of claws with heel horn erosion (HHE) increased significantly in all three SARA groups over the hoof trimming visits, whereas no statistically significant differences were detectable among the three SARA groups, but distinctly higher HHE prevalences were observed in SARA groups moderate and severe than in the light SARA group (Table 4). Within the severe SARA group, a significant difference in HHE prevalence was found between hoof trimming visits 1 and 2 (*p* = 0.040), and between hoof trimming visits 1 and 3 (*p* = 0.038).

The highest prevalence of digital dermatitis (DD) in the 24 first-lactation Holstein cows during the observation period was 3.1% before calving and at 70 DIM (Table 4). No statistically significant differences in DD prevalence could be detected across the three SARA groups or within the SARA groups during the three hoof trimming visits.

### 3.4. Cow Claw Scores

The evaluation of the CCS values related to the individual claw lesions showed similar results to those presented in Section 3.3 regarding the evaluation of the prevalence of the individual claw lesions. Even with this methodology (CCS values of individual claw lesions), there were no significant differences between the three SARA groups. Again, though, statistically significant differences were observed for HHE in the light SARA group between hoof trimming visits 1 and 2 (*p* = 0.002) and between hoof trimming visits 1 and 3 (*p* = 0.005), and in the severe SARA group between visits 1 and 2 (*p* = 0.006) and between visits 2 and 3 (*p* = 0.005). Further, statistically significant differences were observed for double soles in the light SARA group between hoof trimming visits 1 and 3 (*p* = 0.040), and for WLL in the severe SARA group between hoof trimming visits 2 and 3 (*p* = 0.017).

**Figure 4:**
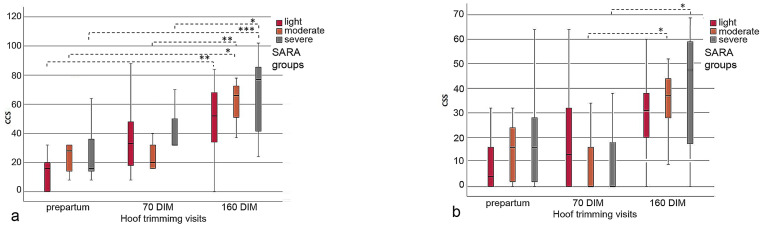
(**a**,**b**) Boxplot graphs illustrating the increase in CCS values from hoof trimming visit 1 to visit 3 in the cows of the SARA groups light, moderate and severe; the left boxplot (**a**) includes the data of all claw lesions; in the right boxplot (**b**) the proportional data of heel horn erosion (HHE) were omitted, which clearly scaled down the CCS values (see the y axis); *–***: indicates significant differences between hoof trimming visits.

The sum of CCS in all three SARA groups increased over the observation period from before calving to three months after the end of the feeding study (160 DIM), assuming that there was a tendency for deterioration in claw health. Further, the CCS values in the severe SARA group were clearly higher compared with those in the SARA groups light and moderate (Figure 4a,b; Table 5). At hoof trimming visit 3, the mean CCS values were higher in all three SARA groups compared to visit 1. No SARA group showed statistically significant differences (*p* = 0.245) from any other group within the three hoof trimming visits. However, statistically significant differences in the CCS values were found when comparing hoof trimming visit 1 with visit 3 (*p* = 0.005) in the light SARA group, comparing visit 1 with visit 3 (*p* = 0.010) and visit 2 with visit 3 (*p* = 0.009) in the moderate SARA group, and visit 1 with visit 3 (*p* = 0.001) and visit 2 with visit 3 in the severe SARA group (*p* = 0.037) (Figure 4a).

Similar results were observed when the CCS values were calculated without the proportional data of HHE (Table 5), because HHE is not considered laminitis-related. No statistically significant differences were found across the three SARA groups at each of the three hoof trimming visits (*p* = 0.889). Nevertheless, there were statistically significant differences when comparing the CCS values of hoof trimming visit 2 with those of visit 3 (*p* = 0.020) in the moderate SARA group, and when comparing the CCS values of hoof trimming visit 2 with visit 3 (*p* = 0.007) in the severe SARA group (Figure 4b).

## 4. Discussion

The effects of high-concentrate feeding and SARA on the claw health of dairy cows has been the subject of numerous studies [18,19,22,23,29,42,43,44]. Contrasting those reports, wherein either an oral stomach tube or ruminocentesis was used to determine rumen pH, in the present study, continuous measurement of rumen pH was performed throughout the study duration of 91 days at 10-min intervals using a sensor placed in the reticulum [45,46]. Another methodological difference in the present experiment compared to previous studies [17,18,19,22,29,44,47] is the definition of SARA. Here, we have determined the number of SARA days, which is only possible by using continuous, and therefore sensor-based, pH measurements [45,46]. According to the literature, a SARA day is defined when the pH value measured by a rumen sensor is below 5.8 for more than 330 min within a 24-h period [28]. Since the duration of SARA is of great importance in defining the severity grade of the acidotic challenge, additional to a lowered rumen pH value [15,27,28,48], the number of SARA days was set in relation to the total duration of the feeding study, and three SARA severity groups were subsequently defined.

The 24 first-lactation Holstein cows were fed a TMR during the feeding study, except during their first week after calving, when the proportion of concentrate was gradually increased from 30% to 60% of the ration DM. This was practiced to avoid the risk of acute rumen acidosis. Lack of adaptation to starchy rations is reported as one of the main risks for rumen acidosis in early lactation, and this is especially true for first-lactation cows [17,18,19,25,44,47].

To stimulate SARA conditions, the concentrate of the close-up and early lactation rations consisted mainly of barley (> 60% of the concentrate). Barley was chosen as it is fermented very quickly in the rumen and, in contrast to corn grain, contains large amounts of rumen-degradable starch. In our experiments, the barley was ground to pass a 3 mm screen using a hammer mill. This results in the formation of large quantities of short-chain fatty acids in the rumen, increasing the risk of SARA when large amounts are fed [20,49,50], as with the SARA diet in the present study.

Yet, despite being fed the same SARA diet, cows responded differently; as many as fifteen cows had >30% SARA days throughout the SARA period, while nine cows had less than 11% SARA days. Thus, as anticipated, the number of days on which rumen pH was below 5.8 for more than 330 min per 24 h [28] varied widely among the 24 cows. The exact mechanisms behind the different response of cows despite being fed the same high-concentrate ration are not yet clear. However, SARA-susceptible and SARA-resistant cows are often reported in the literature [27,29]. This study provides novel insights into the claw health of first-lactation cows exhibiting varying degrees of subacute ruminal acidosis (SARA), while being fed the same high-concentrate diet.

Interestingly, the evaluation of LCS registered at two-week intervals showed a slightly higher mean LCS for SARA groups moderate and severe in contrast to the light SARA group, but there were no statistically significant differences. Only LCS 1 to 3 were observed throughout the duration of the feeding study. However, a statistically significant difference was observed when comparing the lameness incidences of cows in the light SARA group with those of the severe SARA group (3.2% vs. 14.3%). Suspected triggers of pain for these lameness episodes encountered were double soles, WLL, acute stages of DD, and one single sole ulcer. The effective management practices implemented in this farm and in this study, which included functional hoof trimming before the first calving, as well as regular locomotion monitoring every two weeks and prompt treatment of lame cows [51,52,53], can be regarded as decisive factors for the absence of severe (LCS 4, 5) and minimal cases of moderate lameness (LCS 3) throughout the entire feeding trial. Gait monitoring of all cows in the dairy herd at two-week intervals and immediate examination and professional treatment of cattle classified only as mildly lame (LCS 2) is recommended as a very efficient preventive measure for the development of lameness in general, and particularly for the occurrence of moderate and severe locomotion scores [2,3,52,54].

In the 24 first-lactation cows, the mean lameness incidence during the first 70 DIM was very low, at 7.7%, comprising only LCS 2 and LCS 3. Several studies have shown that in first-lactation cows, the prevalence of lameness and CHDL with about 12.8% [55] is significantly lower than in multiparous cows, and that lameness prevalence in cows steadily increases with each lactation [53,55,56,57]. This is because the claws of first-lactation cows bear less weight than those of multiparous cows due to their lower body weight. On the other hand, the claws of heifers and first-lactation cows have not yet been exposed to unilateral pressure loads and negative environmental influences such as hard floors, humid housing conditions, and metabolic and thus laminitis-related events for as long as the claws of multiparous cows [8,55,56,57]. The mean lameness incidence of 7.7% without LCS 4 and 5 scores, as observed in this study, is well below ≤10%, which is reported as the norm key indicator for lameness prevalence in well-managed dairy herds [6,58,59].

In the present study, functional hoof trimming was performed at intervals of approximately four and three months, which is an unusually short interval compared to common practice in many dairy herds [60,61,62]. This management measure could also be cited as an explanation for the low prevalence of lameness and claw lesions in the 24 first-lactation cows. In the context of professional hoof trimming, the usually significantly higher heel height of the lateral hind claw is adjusted to the heel height of the medial claw, if this is at least ≥ 3 cm. This creates an approximately equal load distribution between the lateral and medial claw, but only for a period of a few weeks [60,61,62,63]. Improperly performed hoof trimming, as well as hoof trimming performed at excessively long intervals (≥six months), significantly increases the risk of pressure-related CHDLs, which are largely identical to laminitis-related claw lesions [1,31,61,64].

Since this study investigated the effect of SARA severity on claw health, it was rational to determine the prevalence of claw lesions specifically at the claw level (eight claws per cow), and not at the animal level. With the latter method, a claw lesion is only counted once, regardless of how many claws the lesion was diagnosed in [62,65]. Sole hemorrhages and double soles, and other CHDLs, develop in the context of subclinical, subacute, or acute laminitis when hemorrhage and extensive bruising of the corium occur as the pedal bone descends in the horn capsule [8,9,23,30,31]. Sole hemorrhages and double soles become externally visible on the sole surface about six to eight weeks after they occur, as the horn growth slowly advances from the innermost layers of horn cells to the outer sole horn surface. This process allows for the detection of these conditions through external examination [8,10,30]. This time-delayed hemorrhaging and bruising of the solar corium on the outer surface of the sole horn was the reason why all claws of the cows were examined even three months after study completion. Pressure on the corium vessels after pedal bone sinking is frequently exacerbated by concrete floors, prolonged standing time, and excessively long hoof trimming intervals [61,66,67,68,69]. In this context, it is important to note that the cows in this study were housed on rubber-matted floors and not on hard surfaces with flat concrete or concrete slatted floors, as is very common in dairy farms. Rubber-matted walkways provide a softer, compliant surface, thus reducing pressure damage to the claw corium [67,69]. In addition, the cows used in this study also had access to a well-bedded deep straw area and comfortable and well-maintained cubicles. All these circumstances, which are beneficial to claw health, lead to a reduction in standing time and thus overload on the sole corium [7,68]. In this context, it must be indicated that all the cows experienced the same environmental conditions throughout the entire observation period.

The prevalence of concave dorsal walls, which is considered a characteristic feature of chronic laminitis because they develop only when subclinical, subacute, or acute laminitis has resulted in sinking of the pedal bone within the horn capsule [8,30], increased to about the same extent in all three SARA groups when examined at the hoof trimming visits. However, it is noteworthy that no concave dorsal walls were observed in any cow at the first hoof trimming visit before calving, whereas at hoof trimming visit 3, the mean prevalence was between 23.4% and 28.1% in all three SARA groups, so that statistically significant differences for the prevalence of concave dorsal walls could be noticed between all the three visits in all three SARA groups.

The prevalence of WLL clearly increased over the observation period, with a prevalence of 18.7% in cows in SARA groups moderate and severe. For the severe SARA group, there was a statistically significantly higher prevalence between hoof trimming visit 1 versus visits 2 and 3 (i.e., three months after SARA challenge), indicating a delayed effect of SARA severity on claw health. Previously, a much higher prevalence of WLL at 92% has been reported in first-lactation cows [40] at the claw level, and at 87.1% in Austrian Fleckvieh heifers at the animal level [35]. In addition to feeding, other contributing factors such as excessive mechanical and traumatic stress may be causative for the development of WLL and sole hemorrhages in first-lactation cows [9,32,66]. Since the cows were integrated into the remainder of the herd at the farm after the end of the feeding study, environmental conditions in this barn compartment as well as regrouping may have also contributed to the increased prevalence of WLL [70]. The remainder of the dairy herd also had access to walkways with rubber mats, as well as to a concrete run and to concrete floors in the milking parlor waiting area. In this context, it is important to note that the prevalence of WLL is generally significantly higher in cows housed in loose housing systems than in tie stalls [41,57,71,72].

Across all three SARA groups, the prevalence of HHE was found to be the highest among all other claw lesions, ranging from 28.5% to 87.5%. Notably, at the first hoof trimming visit conducted prior to calving, the presence of HHE was already remarkably high, surpassing that of any other claw lesion. The prevalence and severity of HHE is mainly dependent upon hygienic housing conditions. Humid- and manure-soiled walking and lying areas promote the occurrence of HHE [67,73]. Thus, HHE is not considered a laminitis-related claw horn lesion [8,10]. Therefore, the geometric severity score values in the CSS evaluation for the three SARA groups were also calculated without inclusion of HHE values. HHE is not usually associated with lameness, and some HHE prevalence is unavoidable even in cows kept in well-managed loose housing [6,59,73]. The cows used in this study had dry and comfortable deep straw-bedded areas and cubicles available during the entire study period. It could also be argued that this arrangement explains the finding that there were no significant differences among the three SARA groups regarding HHE prevalence.

During the entire duration of the study, only one sole ulcer (1.56%) was diagnosed. The prevalence of sole ulcers in the 24 first-lactation cows was thus at a similarly low level to the 1.4% reported in a study of 139 Austrian Fleckvieh heifers [35].

The prevalence of DD was also not statistically significantly different among the three SARA groups, or at the three hoof trimming visits within the SARA groups, and was also very low, at only 3.1%. This low DD prevalence is striking; other authors have reported higher DD prevalence in first-lactation cows compared to multiparous cows [74,75]. As an explanation for the very low DD prevalence in this study herd, the good housing conditions, the frequent scraping of walkways, the regular locomotion scoring at two-week intervals and the immediate and adequate treatment of animals identified as lame can be cited, i.e., all of these are effective preventive measures, as recommended for herds with endemic DD infection [73,74,75].

Using the numerical claw health score called the Cow Claw Score, the claw health of individual cows can be compared more easily than by comparing the prevalence of individual claw lesions [35,36,76]. This CCS value can be calculated from all documented claw lesions and their three severity scores on all eight claws per cow by geometric weighting. Therefore, so-called “alarm diseases”, i.e., claw lesions that are always associated with pain and therefore lameness [6], are weighted significantly more heavily [35,36,41]. However, the CCS values did not show a statistically significant difference across the three SARA groups, which can be explained by the low number of animals and the overall low prevalences of the individual claw lesions. However, higher CCS values were observed in the severe SARA group than in the SARA groups light and moderate at all hoof trimming visits, albeit without statistical evidence. This could be attributed to the fact that early effects on claw health also result in SARA-susceptible cows. Another evaluation of the results shows that there were statistically significant differences for CCS values within the three SARA groups when comparing hoof trimming visit 1 with visit 3 in the light SARA group and comparing trimming visit 1 with visit 2 and visit 1 with visit 3 in the SARA groups moderate and severe, respectively. The CCS means ranged from 13.00 in the light SARA group before calving to 66.75 in the severe SARA group at hoof trimming visit 3. A claw health score per cow (CCS) ≤ 35 is thereby indicated as a good value [35,36,76]. The exclusion of proportional data of HHE, which is not considered to be laminitis-related [8,10], in the calculation of CCS values yielded comparable outcomes. Notably, statistically significant differences were observed between CCS values at hoof trimming visit 2 and visit 3 in the moderate and severe SARA groups.

Other favoring factors, which are not directly SARA-related but play an additional role in the development of CHDL, include the parturition process itself, unavoidable energy and nutrient deficits in early lactation, and an associated thinning of the digital fat cushions in the claws, and thus a reduction in their shock-absorbing properties, as well as body weight gains of cows in the medium term [1,8,12,43]. However, despite unavoidable energy and nutrient deficits in cows during the 3–4 weeks after calving, the first-lactation cows in this study had no clinical health disorders, such as ketosis, except the reported lameness episodes.

A few limiting aspects must be mentioned in terms of the present study. These include the small number (24) of animals studied, which can be cited as one explanation for the lack of statistically significant differences across the three SARA groups regarding the prevalence of claw lesions and CCS values. Further, in this study, we did not have a negative control group, i.e., a group in which no cow had SARA. However, it has to be emphasized that the cows were carefully preselected for comparable genetic background, performance, age and body size, and this considerably reduced variability. Additionally, the continuous monitoring of the ruminal pH allowed for the accurate determination of SARA in all 24 cows, and the 24 cows had already been kept together in a group for five months prior to calving, which reduced the transition stress together with birth [70]. On many dairy farms, heifers are often not integrated into the dairy herd until immediately after calving, resulting in ranking fights [70]. This stress around the time of parturition and difficulties in adjusting to a new herd are important reasons for the occurrence of laminitis, along with feed conversion and the increased risk of other acute general diseases during this critical period [11,15,21,44]. Moreover, in many farms, changes of the walking surface frequently occur for the animals when they are moved to the lactating herd, which was also not true for the cows in this study.

The feeding study took place in groups at different seasons of the year, since the 24 heifers did not all calve at the same time, but rather their calving dates extended from March 2021 to September 2021. Although the distribution of the cows in different SARA groups was not affected by the calving season, the season influences the ambient temperature of the cows and thus the risk of heat stress [16,22,68]. Prolonged standing time due to heat stress, but also due to overcrowding and inadequate lying surfaces, enhances the negative effects of laminitis on claw health because it further increases the load on the claw corium [7,68,69]. In this study, temperature and humidity data were not recorded.

Another important explanation for the lack of statistically significant differences in the prevalence of individual claw lesions and CCS values between the three SARA groups could be the relatively short period of 70 days in which the starchy ration was fed. In practice, starchy rations are usually fed for much longer, and often over several lactations. Thus, cows frequently exhibit SARA over a disparately longer period [14,20,77]. This argument is also supported by the risk analysis performed, which showed that in cows, for each day under SARA, the probability of becoming lame increased by 2.52%. Repeated episodes of SARA can lead to the development of subacute or subclinical laminitis, which can ultimately progress to chronic laminitis characterized by a claw concave dorsal wall after several months [8,30]. Chronic laminitis and repeated laminitis bouts lead to morphological changes in the claw, with negative effects on horn quality and weight distribution on the claws. Thus, the risk of developing laminitis-related CHDL increases when feeding highly fermentable diets over a prolonged period and in multiple lactations [42,43,44,56,57].

## 5. Conclusions

Although the cows in the severe SARA group tended to have poorer claw health than those in the light SARA group, this difference could not be statistically validated in terms of overall claw lesion prevalence, LCS or CCS. However, regarding the lameness incidences, we observed statistically significant differences when comparing the light SARA group with the severe SARA group. In addition, data analysis revealed significant differences in the prevalence of double soles and WLL within the severe SARA group during the three hoof trimming visits, with significantly higher prevalences at hoof trimming visit 3. Similar statistical differences were revealed for the CCS values within the three SARA groups when comparing the hoof trimming visits. Thus, the results of this study partly support the established hypothesis. The possible reasons for the minor differences in the prevalences of claw lesions and CCS values across the three SARA groups most likely include the small number of included cows, and particularly the short duration of the feeding study. Based on the results of the risk analysis, it is anticipated that the risk for lameness in first-lactation cows increases after around 40 days under SARA, whereby a high probability (>25%) of becoming lame was estimated when SARA duration exceeded 80 days.

## Figures and Tables

**Table 1:** Composition of close-up ration, SARA ration, and post-SARA ration. ^1^

Components (% of DM Unless Stated)	Close-Up Ration	SARA Ration	Post-SARA Ration
Grass silage	38.0	24.0	37.0
Corn silage	30.0	16.0	20.9
Meadow hay	0.0	0.0	4.3
Wheat straw	0.0	0.0	1.6
Concentrate mixture ^2^	32.0	60.0	0.0
Grain mixture with premix ^3^	0.0	0.0	9.8
Protein supplement ^4^	0.0	0.0	5.5
Dairy concentrate ^5^	0.0	0.0	20.9
*Estimated composition*			
Dry matter (% as is)	40.3	43.3	38.8
Crude protein	14.3	17.0	14.8
Starch and sugars	27.9	35.1	21.1
Neutral detergent fiber	39.2	29.6	45.2
Net energy of lactation (MJ/kg DM)	6.5	7.2	6.4

^1^ Close-up (3 weeks prepartum until calving day), SARA diet (fed until 70 DIM), and post-SARA ration (>70 DIM) were fed as total mixed rations (TMR).^2^ Concentrate mixture for close-up ration contained: 62% barley grain, 20% rapeseed meal, 9% corn meal, 4% soybean meal with 44% crude protein, 4% vitaminized mineral feed, and 1% molasses. Concentrate mixture for SARA ration contained: 61.5% barley grain, 16% rapeseed meal, 8% corn meal, 10% soybean meal with 44% crude protein, 3.5% vitaminized mineral feed, and 1% molasses; both concentrate mixture were fed in pelleted form.^3^ Grain mixture with premix contained: 50% corn meal, 20.8% barley grain, 20.8% wheat meal, 3.3% limestone, 5% vitaminized mineral feed (Rindavit TMR 11 ASS-Co+ATG, Schaumann GmbH).^4^ Protein supplement was Rindastar39 (Schaumann GmbH) with 39% crude protein.^5^ Dairy concentrate was pelleted KuhKorn Kompakt19 (Garant Tiernahrung) with 19% crude protein. DM: dry matter.

**Table 2:** Overview of claw lesions, their codes, the three severity scores per lesion as recorded by the electronic documentation system and assigned geometric severity scores.

Term of Claw Lesion	Lesion Code	Severity Score	* Geometric Severity Score
Asymmetric claw	AC	1	0
Concave dorsal wall	CD	1	5
		2	10
		3	20
Corkscrew claw	CC	1	4
Digital dermatitis M1	DD-M1	1	16
Digital dermatitis M2	DD-M2	3	64
Digital dermatitis M3	DD-M3	1	16
Digital dermatitis M4	DD-M4	1	8
Digital dermatitis M4.1	DD-M4.1	2	32
Interdigital dermatitis	ID	1	8
Double sole	DS	1	4
		2	8
		3	32
Heel horn erosion	HHE	1	2
		2	4
		3	16
Horn fissure vertical	HFV (HFA, HFD)	1	8
(axial, dorsal)		2	16
		3	64
Horn fissure horizontal	HFH	1	8
		2	16
		3	64
Interdigital hyperplasia	IH	1	8
		2	16
		3	64
Interdigital phlegmon (foot rot)	IP	1	64
		2	96
		3	128
Scissor claws	SC	1	0
Swelling of coronet and/or bulb	SW	1	16
		2	32
		3	64
Sole hemorrhage (circumscribed/	SH	1	4
diffuse)		2	8
		3	16
Sole ulcer	SU	1	32
		2	64
		3	128
Bulb ulcer	BU	1	32
		2	64
		3	128
Toe ulcer	TU	1	32
		2	64
		3	128
Toe necrosis	TN	3	128
Thin sole	TS	2	24
White line lesion (separation)	WLL	1	16
White line abscess	WLA	2	64
	WLA	3	128
DD-associated bulb ulcer	DD-BU	3	128
DD-associated horn fissure	DD-HF	3	128
DD-associated interdigital hyperplasia	DD-IH	3	128
DD-associated sole ulcer	DD-SU	3	128
DD-associated toe ulcer	DD-TU	3	128
DD-associated toe necrosis	DD-TN	3	128
DD-associated white line abscess	DD-WLA	3	128

* Geometric severity score calculation as is used in the “*Klauenmanager”* documentation program according to reports in the literature [35,36,40,41] and recent extensions together with Swiss researchers (A. Steiner, C. Syring).

**Table 3:** LCS means of cows in the three SARA groups during the study period.

SARA Group ^1^	LCS Mean	SD	Min/Max
Light	1.03	0.18	1/2
Moderate	1.07	0.32	1/3
Severe	1.14	0.35	1/2

^1^ For SARA classification see Figure 1. SD: standard deviation. min/max: minimum/maximum values. LCS 1 (locomotion score 1): not lame.

**Table 4:** List of prevalence values in percentage (at claw level) of recorded claw lesions in the three SARA groups before the start of the study (8 weeks before calving), at the end of the SARA diet (70 DIM) and three months later (160 DIM) after the SARA diet was discontinued.

Claw Lesion Type (Code)	SARA Group ^1^	Before Calving	At 70 DIM	At 160 DIM
Interdigital hyperplasia (IH)	L	0.0	0.0	0.0
	M	0.0	0.0	0.0
	S	0.0	1.6	0.0
Sole ulcer (SU)	L	0.0	0.0	0.0
	M	0.0	0.0	0.0
	S	1.6	0.0	0.0
Horn fissure (HF)	L	0.0	0.0	0.0
	M	0.0	0.0	0.0
	S	3.1	0.0	0.0
Digital dermatitis (DD-M2)	L	0.0	3.1	0.0
	M	3.1	0.0	0.0
	S	0.0	1.6	1.6
Corkscrew claw (CC)	L	0.0	6.2	6.2
	M	0.0	0.0	0.0
	S	0.0	0.0	0.0
Sole hemorrhage (SH)	L	3.6	3.1	0.0
	M	0.0	1.6	4.7
	S	0.0	6.2	7.8
Double sole (DS)	L	0.0	6.2	12.5 *
	M	1.6	0.0	0.0
	S	1.6	0.0	0.0
Concave dorsal wall (CD)	L	0.0	3.1	25.0 *
	M	0.0	3.1	23.4 *
	S	0.0	3.1	28.1 *
White line lesion (WLL)	L	8.9	9.4	12.5
	M	14.1	6.2	18.7
	S	12.5	3.1	18.7 *
Heel horn erosion (HHE)	L	28.7	71.9	59.4
	M	62.5	68.7	84.4 *
	S	37.5	87.5 *	84.4 *

^1^ SARA groups are shown in Figure 1; L = light SARA group (cows experiencing SARA in <11% of the experimental days); M = moderate SARA group (cows experiencing SARA in >11 to <30% of the experimental days); S = severe SARA group (cows experiencing SARA in >30% of the experimental days); DD-M2: acute DD lesion; *: indicates significant differences between hoof trimming visits.

**Table 5:** CCS means of the cows of the three SARA groups at the three hoof trimming visits; the numbers in parentheses indicate the mean CCS values without inclusion of the proportional data of heel horn erosion (HHE).

SARA Group ^1^	Before Calving	At 70 DIM	At 160 DIM
Light	13.0 (9.0)	36.8 (19.2)	49.0 (29.7)
Moderate	28.6 (18.5)	26.2 (8.2)	61.7 (34.9)
Severe	25.5 (19.5)	37.2 (9.2)	66.7 (39.7)

^1^ SARA groups are shown in Figure 1.

## Data Availability

Data are contained within the article.
